# Facing the future

**DOI:** 10.1098/rsbl.2023.0361

**Published:** 2023-09-27

**Authors:** David Beerling

**Affiliations:** School of Biosciences, University of Sheffield, Sheffield S10 2TN, UK

*Biology Letters* is a Royal Society journal focused on the rapid publication of short high-quality research articles, reviews and opinion pieces across the biological sciences. We are also a Plan S compliant (https://royalsociety.org/journals/open-access/) Transformative Journal. We continue to grow and innovate to provide the best service to our authors and readers in the face of a challenging publishing landscape.

The team at *Biology Letters* is committed to offering an excellent publishing experience for authors from maintaining a painless submission process to highly competitive publishing median times, with an average of 26 days from receipt to first decision and 15 weeks from first submission to publication. These turnaround times are testament to the hard work of our 90 strong international Editorial Board, comprised of expert Board Members, and Handling, Reviews and Preprint Editors. All these experts are efficiently organized by our excellent staff, Surayya Johar and Raminder Shergill, based in the Editorial Office at the Royal Society, London. But we are not complacent, and continually renew our Editorial Board to ensure it includes the best scientists to advise us on decision-making across our diversity of subject areas and to ensure it reflects gender and geographical diversity. If you are interested in joining our Editorial Board please contact the Editorial Office.

We are committed to increasing the accessibility of research and ensuring that it is communicated as rapidly as possible. To accelerate this process, we encourage researchers to deposit pre-publication versions of articles in appropriate preprint repositories such as bioRxiv (https://www.biorxiv.org/), paleorXiv and EcoEvoRxiv (https://ecoevorxiv.org/). Over the past year, our Preprint Editor, Catherine Talbot, has been recruiting more people to join her team and expand the subject area coverage. Again, please do contact the Editorial Office if you are interested in learning more about joining our preprint team.

Staying with the preprint landscape, I would like to take this opportunity to highlight that *Biology Letters* supports submission of manuscripts directly from bioRxiv. This is in-line with our sister Royal Society journals (e.g. *Open Biology*, *Proceedings B* and *Journal of the Royal Society Interface*). It means authors can transfer their manuscript information and metadata without having to re-upload files. We encourage authors to cite the preprint version in the accepted article. Authors are also permitted to cite other preprints, provided they are clearly identified as such.

As we embrace future developments to widen participation in scientific publishing, there is a need to be mindful of the advent of new artificial intelligence (AI)-assisted technology, such as ChatGPT. Indeed, there are now examples of published articles written using ChatGPT, and most recently, of scientists taking it a step further by producing a research paper in less than an hour with ChatGPT as the ‘co-pilot’. These endeavours are exploring the frontiers of what AI technology may offer scientists in the future. Perhaps most importantly, they raise awareness of the need for discussions about its benefits and risks. Currently, in *Biology Letters*, authors must declare where they have used AI in their manuscript. Declaring the use of these technologies supports transparency and trust. Please do familiarize yourselves with our current AI policy when you are submitting to the journal.

An important element of the *Biology Letters* publishing model is our ability to assemble and publish topical collections of articles as Special Features. These collections follow from authors' suggestions approved by the Editorial Board. In recent years, Special Features collections have included Ocean Acidification (2017), Effect of Sea Ice on the Arctic Biota (2018), Blue Carbon (2019) and Ecological Resilience (2021). Last year's Special Feature on the causes and consequences of worldwide Insect Decline could not be more topical. These articles aimed to increase knowledge of potential causes and consequences of this decline. Rather than merely focusing on additional evidence for insect population declines, the authors provided a better mechanistic understanding of the causes for, and consequences of, insect decline. We welcome and look forward to suggestions for future topics.

As leading scientists, our authors often make the headlines with their research discoveries reported in *Biology Letters*. Earlier this year, Warren Booth and colleagues reported the first case of a crocodile who made herself pregnant, as identified at a zoo in Costa Rica. The discovery made headlines around the world. The phenomenon, known as parthenogenesis, occurs in birds, insects, fish and other reptiles but had not previously been reported in crocodiles. From Booth's comment to the BBC about the work, it is easy to see why it attracted press interest: ‘The fact that the mechanism of parthenogenesis is the same in so many different species suggests that it is a very ancient trait that has been inherited throughout the ages. So this supports the idea that dinosaurs could also reproduce this way.’

As always, I would like to thank our talented and dedicated authors, readers and reviewers for all their hard work over the past year. The success of the journal reflects their efforts, together with those of our Editorial Board and publishing team.

In closing, I would like to recommend an excellent publishing blog (https://royalsociety.org/blog/2023/06/biology-letters-has-your-research-covered/) on the Royal Society's site written by our own Surayya Johar who caught up with some of our authors whose images made it onto the front cover of *Biology Letters* to find out what happened next.

